# High-throughput ligand profile characterization in novel cell lines expressing seven heterologous insect olfactory receptors for the detection of volatile plant biomarkers

**DOI:** 10.1038/s41598-023-47455-4

**Published:** 2023-12-08

**Authors:** Katalin Zboray, Adam V. Toth, Tímea D. Miskolczi, Krisztina Pesti, Emilio Casanova, Emanuel Kreidl, Arpad Mike, Áron Szenes, László Sági, Peter Lukacs

**Affiliations:** 1 Plant Protection Institute, Centre for Agricultural Research, Martonvásár, Hungary; 2 TetraLab Ltd., Budapest, Hungary; 3 Department of Biochemistry, Eötvös Loránd University, Budapest, Hungary; 4 Department of Pharmacology, Center of Physiology and Pharmacology, Comprehensive Cancer Center, Medical University of Vienna, Vienna, Austria; 5 Department of Pathology, University of Veterinary Medicine, Budapest, Hungary; 6 Agricultural Institute, Centre for Agricultural Research, Martonvásár, Hungary; 7 Present Address: Novartis AG, 6336 Langkampfen, Austria

**Keywords:** High-throughput screening, Fluorescent proteins, Ligand-gated ion channels, Screening, Olfactory receptors, Diagnostic markers

## Abstract

Agriculturally important crop plants emit a multitude of volatile organic compounds (VOCs), which are excellent indicators of their health status and their interactions with pathogens and pests. In this study, we have developed a novel cellular olfactory panel for detecting fungal pathogen-related VOCs we had identified in the field, as well as during controlled inoculations of several crop plants. The olfactory panel consists of seven stable HEK293 cell lines each expressing a functional *Drosophila* olfactory receptor as a biosensing element along with GCaMP6, a fluorescent calcium indicator protein. An automated 384-well microplate reader was used to characterize the olfactory receptor cell lines for their sensitivity to reference VOCs. Subsequently, we profiled a set of 66 VOCs on all cell lines, covering a concentration range from 1 to 100 μM. Results showed that 49 VOCs (74.2%) elicited a response in at least one olfactory receptor cell line. Some VOCs activated the cell lines even at nanomolar (ppb) concentrations. The interaction profiles obtained here will support the development of biosensors for agricultural applications. Additionally, the olfactory receptor proteins can be purified from these cell lines with sufficient yields for further processing, such as structure determination or integration with sensor devices.

## Introduction

The composition of volatile organic compounds (VOCs) in airborne emissions provides valuable information for various applications, including the detection of explosives or toxic materials^1^ as well as the differentiation between healthy and diseased biological samples based on specific VOC blends^2^.

In the animal kingdom, VOCs are bound and recognized by odorant or olfactory receptors (ORs), although several other peptide molecules such as Odorant Binding Proteins (OBP) are also capable of specific VOC recognition.

It is due to these receptors that animals, such as drug-sniffing dogs, can detect and trace marker volatiles with unprecedented sensitivity. Mammals have several hundred to a thousand ORs, which are G-protein-coupled receptors (GPCRs)^3^. Numerous research programs have focused on the development of biosensors based on such mammalian receptors. However, due to their action mechanism, the receptors themselves are not sufficient for odorant perception, and additional signal transduction molecules are required for their proper function^4^.

In contrast, insect ORs have a substantially different structure and mode of operation. For insects, too, odor perception is critical for feeding, oviposition, mate recognition, and predator avoidance^5^; therefore, insects are also able to sense relevant odorants at minute concentrations. Moreover, they possess a much narrower receptor repertoire compared to mammals, and—unlike GPCRs—do not need additional components for odorant recognition^6^.

Insect ORs are typically assembled from two types of proteins: the tuning OR, which is responsible for VOC binding and ligand-specificity, and the Olfactory Receptor Co-receptor (ORCO^7^) which is essential for proper OR folding and function. While the amino acid sequence of OR proteins is very diverse, that of ORCO proteins is highly conserved even in diverse species. In many cases OR and ORCO protein combinations from different species can form functional channels. Cryo-EM structure determination on the ORCO protein from a parasitic fig wasp (*Apocrypta bakeri*) revealed a homotetrameric structure in the resulting autonomous cation channel^8^.

Regarding their action mechanism insect ORs predominantly work as ligand-gated ion channels^9,10^, although G-protein-mediated signaling was also indicated in several studies^9,11–14^. VOC ligand binding induces channel opening and an influx of cations. Channel permeability is much higher for monovalent (e.g., Na^+^, K^+^, and Cs^+^) than for divalent (Ca^2+^ and Mg^2+^) cations, and permeability ratios also depend on the identity of the OR^8^. Under physiological conditions, ORs are localized in the dendrites of olfactory sensory neurons, where they depolarize the membrane upon ligand binding. This depolarization activates voltage-gated ion channels and changes the firing rate of the neuron^15^.

Using the antenna of the fruit fly it was possible to distinguish between the smell of healthy and cancer cell lines based on their different OR activation patterns^16^. The receptor repertoire was mapped earlier for *Drosophila melanogaster*^17^. In vivo measurements, however, are not compatible with a sensor for the analysis of multiple samples. An olfactory panel consisting of several ORs selected for the specific sensing of the target molecules could work effectively as a biosensor.

Several research groups conducted experiments with heterologously expressed or purified ORs linked to sensor devices, but a complex insect receptor panel has not yet been developed. As a first step to this direction the ligand profiles of the sensory proteins must be characterized.

Here we present an improved method for the generation of OR-expressing cell lines in which odorant binding is easy to follow by GFP fluorescence change without the need of loading the cells with a dye. As an example of the methodology, we generated seven stable cell lines in HEK293 cells all expressing a different *Drosophila* OR, ORCO, and GCaMP6 as a genetically encoded calcium ion sensor protein. Using these cell lines as an olfactory panel, the concentration–response profile of a large set of odorants was mapped here for the first time. The set of 66 tested VOC molecules was compiled in relation to our research on plant- and plant pathogen-derived VOCs^18^. VOC-specific cell responses were determined with a high-throughput microplate reader assay over a concentration range of three orders of magnitude. The new OR cell lines and hitherto untested plant disease-related volatiles represent original contributions to the present panel and ligand list of the ORs examined.

## Results and discussion

### Generation of the stable OR cell lines

In vivo measured ligand profile data derived from the DoOR database (http://neuro.uni-konstanz.de/DoOR/default.html)^19,20^ were used to select 12 ORs, which can be appropriate to detect the pathogen-related VOCs relevant for our study. To assemble a novel cell-based olfactory panel, 11 stable cell lines were successfully generated which expressed different *Drosophila* ORs and the fluorescent calcium ion indicator protein GCaMP6^21^ in HEK293 cells. Out of these cell lines, seven were responsive to the respective OR-specific VOCs. Using these functional lines we constructed an olfactory assay in which binding of the examined odorants can be quantified by a fluorescence signal.

Four different proteins were expressed in each of the cell lines: the respective ORs, the ORCO universal co-receptor, the mCherry fluorescent protein as a marker for OR expression, and the GCaMP6 fluorescent calcium ion indicator protein. To ensure stable and high-level expression, bacterial artificial chromosome (BAC) vectors containing an approximately 70-kb long open chromatin region (BAC^Rosa26^) were used to deliver the transgenes into the cells.

In comparison to conventional expression vectors, the use of a full-length, 210 kb BAC^Rosa26^ expression vector could increase up to nine-fold the yield of heterologous secreted proteins^22^. It also helped to express high amount of recombinant sodium channels in CHO cells^23^. However, transfection of this large DNA was much less efficient compared to shorter plasmids. Therefore we generated a truncated 70 kb version of BAC^Rosa26^ by homologous recombination and FLP recombinase mediated mutagenesis. (Supplementary Fig. 4).

Transfection efficiency and long-term protein production capacity were tested for this truncated 70 kb BAC^Rosa26^ by GFP expression. Chinese hamster ovary (CHO) cells were nucleoporated with a plasmid, original 210 kb BAC^Rosa26^ and truncated 70 kb BAC^Rosa26^ vector all containing CAG promoter, GFP and Neomycin resistance gene as a selection marker. The ratio of GFP positive cells were approximately 3-times higher 24 h post transfection for truncated 70 kb BAC^Rosa26^ transfected cells (12% GFP positive of living cells) than for original 210 kb BAC^Rosa26^ transfected ones (4.1%) while it was less compared to plasmid transfected cells (17.1%) (Supplementary Fig. 4). The same cell pools were cultured for an additional 2 months in the presence of antibiotic selection pressure. Both BAC^Rosa26^ transfected cell pools showed high GFP intensity levels (Supplementary Fig. 4) with 88.3% GFP positive cells for the original BAC^Rosa26^ and 99.3% of the truncated BAC^Rosa26^ while in the plasmid transfected cell pool only 42.7% of the cells showed GFP fluorescence despite of constant selection pressure.

As the truncated 70 kb BAC^Rosa26^ could ensure high protein expression level but combined with more efficient transfection compared to the original version we used this as an expression vector in this study.

The expression of the OR and mCherry proteins was driven by the strong and constitutive CAG promoter^24^. The remaining two proteins, ORCO and GCaMP6, were expressed as a single fusion protein by linking GCaMP6 N-terminally to ORCO to allow membrane localization. Based on the experience of Corcoran et al.^25^ constitutive ORCO expression can lead to cell death after several weeks. In order to ensure long-term viability of our OR cell lines, the doxycycline-inducible Ptet-T6 promoter^26^ was chosen to drive the expression of the GCaMP6-ORCO fusion protein.

**Figure 1:**
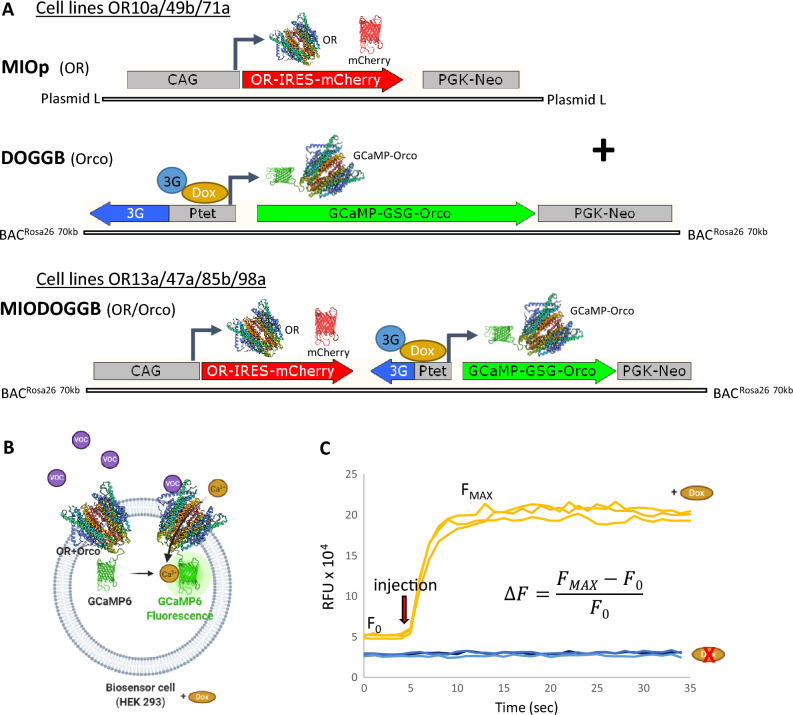
Generation and characterization of OR-expressing cell lines. (**A**) DNA vector constructs for OR cell line production: MIOp plasmid containing the corresponding OR and mCherry, and DOGGB BAC expression vector coding for the GCaMP6-Orco fusion protein were cotransfected to generate OR10a, OR49b and OR71a cell lines (upper panel); MIODOGGB BAC expression vectors containing the corresponding OR and all other components were used to generate OR13a, OR47a, OR85b and OR98a cell lines (lower panel). The inducible Ptet bidirectional promoter only activates GCaMP6-Orco protein expression upon doxycycline (Dox) treatment. (**B**) Detection mechanism of the cell lines: the OR + Orco receptor complex opens upon VOC ligand binding, the resulting Ca^2+^ influx is detectable as GCaMP6-mediated fluorescence. *VOC* volatile organic compound. (**C**) Fluorescence signal intensity change of the OR47a cell line 48 h after doxycycline induction (orange curves) and without doxycycline (blue curves) in response to 50 µM VUAA1. Each curve represents a technical replicate measured in separate wells of a 384-well microplate. *RFU* relative fluorescence unit, *F*_*MAX*_ maximal RFU value during the measurement, *F*_*0*_ average baseline fluorescence, ∆*F* was calculated according to the equation shown.

To test if the arrangement of genetic elements has an impact on gene expression or not, we cloned and transfected them in two different formats. In the OR13a, OR47a, OR85b, and OR98a cell lines the two expression units were physically integrated tandem (Fig. 1A) into BAC^Rosa26^. In this arrangement the Ptet-T6 promoter exhibited no baseline expression. Based on binding experiments with the ORCO-activating artificial ligand VUAA1^27^ there was no GCaMP6-ORCO expression without doxycycline treatment whereas a strong induction was recorded 48 h after doxycycline treatment (Supplementary Fig. 1A). The OR10a, OR49b, and OR71a cell lines, on the other hand, were generated by cotransfection of the same two expression units (Fig. 1A).

In these cell lines, the Ptet-T6 promoter had a substantial baseline activity which was further increased upon doxycycline induction (Supplementary Fig. S1B). In the light of these results, we concluded that tandem integration of the expression units is the method to choose for future cell lines as it prevented uninduced Ptet-T6 promoter activity. Nevertheless, this leaky promoter activity did not hinder the maintenance of these cell lines as their responsiveness was stable during the 60 days monitored (Supplementary Fig. S2). Thus, the previous observation that constitutive ORCO expression was detrimental to long-term cell culture^25^ could not be confirmed for *Drosophila* OR10a, OR49b, and OR71a + ORCO combinations in our experiments. The OR cell lines may have different sensitivity to constitutive ORCO expression because the ion permeability of the channel depends on the OR identity^8^. Alternatively, components in the culture medium may activate the ORs in some cell lines, but not in others, due to their different ligand profiles. In four stable cell lines (OR7a, OR19a, OR69a, and OR47b) none of the tested VOCs could activate the ORs despite the fact that OR7a, OR19a and OR69a showed strong responses to multiple VOCs according to the DoOR database. Similarly to the seven responding cell lines, the mCherry fluorescence signal was strongly visible indicating the presence of at least the OR transcripts as mCherry was transcribed from the same mRNA as the ORs. All cell lines responded to VUAA1 indicating the presence of functional GCaMP6-ORCO fusion proteins. Lack of response of some ORs in heterologous expression systems was reported in several previous studies^17,28–32^ It was shown that translation can be a bottleneck in these cases. Functional OR protein expression can be improved by codon optimization of OR genes instead of using wild type sequences^33^. In our study, we used a codon-optimized version of ORCO, however, we did not optimize any of the OR genes. OR release from the ER and trafficking to the plasma membrane could have been hindered in non-responding cell lines, too. The use of signal peptide tags might help in these cell lines as it was previously shown that their use could significantly enhance the intensity of the Ca^2+^ response of the Drodophila OR47a/ORCO transfected cells in transient expression studies^34^.

Out of the seven responsive OR cell lines reported here only one, OR13a was stably expressed so far in an insect cell line (Sf21)^35^. This and other ORs were expressed transiently either by mRNA injection into *Xenopus* oocytes or by transfection into immortalized cell lines: OR13a^36^, OR10a and OR71a^37,38^, OR47a^9,34,39^, OR49b^39,40^, and OR85b^41,42^. In contrast, all our stable cell lines can be used for months for direct measurements. Moreover, the cell cultures can be scaled up, then cryopreserved and thawed again at any time for subsequent measurements.

### The responsiveness of the OR cell lines to their reference ligands

**Figure 2:**
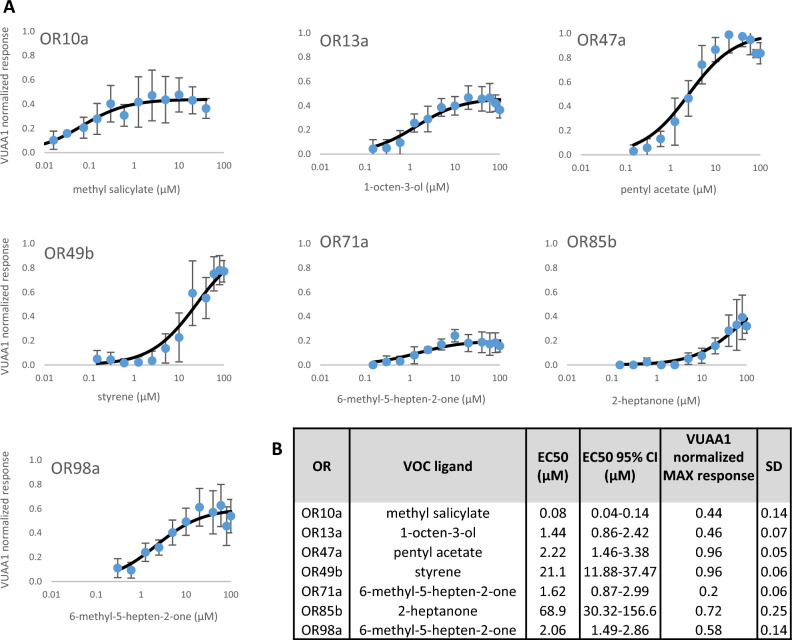
Concentration-dependent response of the OR cell lines to the respective reference VOC ligands. (**A**) VUAA1-normalized response was calculated by dividing VOC response with the average of VUAA1 response on the day of the measurement for the corresponding OR cell line. Concentration–response curves are shown as the average of 5 measurements, error bars represent the standard error (SD). (**B**) EC50 values and maximal VUAA1 normalized responses were calculated from the fitted Hill equation,where the Hill coefficient was constrained to 1 and the maximal response was maximum 1. The calculations and the original traces are available in the supplement.

The functionality of the OR cell lines was verified by the artificial ORCO agonist VUAA1 and at least one OR-specific odorant (Fig. 2). To identify OR-specific reference ligands, two or three strong ligand candidates were chosen based on the DoOR database (http://neuro.uni-konstanz.de/DoOR/default.html)^20^ and tested at 100 μM final concentration. The compound that triggered the highest response was chosen as the reference ligand for the given OR. For four cell lines (OR49b, OR71a, OR85b, and OR98a)—from our plant disease-related ligand list—the strongest agonists were not the ones which were suggested by the DoOR database. We used styrene instead of 2-methylphenol for OR49b, 6-methyl-5-hepten-2-one instead of 4-ethylguaiacol for OR71a and instead of ethyl benzoate for OR98a, and 2-heptanone instead of butyl acetate for OR85b as reference ligands (Fig. 2B, Supplementary Table 2).

We used the GFP-linked calcium ion indicator GCaMP6, in order to measure the response of the cells as a fluorescence signal (Fig. 1B, C).

The maximum ∆F values measured on the OR cell lines were exceptionally high, ranging between 124 and 462 ΔF% in response to 50 μM VUAA1 and 29–339 ΔF% to their reference ligands during long term stability testing (Supplementary Fig. S2), in contrast to 20–60% previously measured in comparable experimental systems^25,35,43^. OR expression level of the cells can show some variation between measurement days. To minimize any VOC response differences due to this variation, the measured ∆F values were normalized to average cell line-specific ∆F values in response to 50 μM VUAA1 measured on the same day and were counted as 1. These VUAA1 normalized ∆F values were used to compare the different receptors and ligands.

We tested for the origin of the Ca^2+^ ions on two cell lines, OR47a and (GCaMP6-GSG)ORCO, with assay buffer lacking Ca^2+^ and assay buffer lacking Ca^2+^ and containing EGTA. Neither of the cell lines responded to the ORCO agonist ligand (Supplementary Fig. S3), indicating that the signal is caused by extracellular Ca^2+^.

The time requirement for the measurement of one VOC sample in the seven OR cell lines was 13 min when measured in triplicates (three wells/sample/cell line). It was possible to measure 16 samples within approximately 3.5 h in a whole 384-well microplate.

### Comparison of ligand sensitivity with previously published OR-expressing cell lines

The concentration-dependent effect of the reference ligands on their cognate cell lines was examined in more detail. Twelve different ligand concentrations were measured on each cell line in an overall 0.01 μM to 100 μM concentration range with 5 repetitions with three technical replicates each time. The half-maximal effective concentration (EC50), and the maximal response compared to 50 μM VUAA1 were calculated (Fig. 2B).

Based on these parameters, a comparative evaluation of the OR cell lines was performed. There are a number of studies in which HEK293 cells have been used for functional characterization of insect ORs^25,33,34,44–46^. In these studies, typical EC50 values and ligand sensitivity of the ORs were very similar to our results, but our system shows a much higher fluorescence intensity change (ΔF) in response to VOC ligands.

In an insect cell line stably expressing OR13a, 1-octen-3-ol evoked concentration-dependent Ca^2+^ signals with an EC50 of 4.33 μM^35^. The effect of the same ligand has been investigated recently on heterologously expressed OR13a receptors of a different fruit fly species (*Bactrocera dorsalis*) in HEK293 cells. An EC_50_ of 11.28 μM in fluorescent measurements, and 1.268 μM in electrophysiology was detected^36^. Our OR13a expressing cells had similar sensitivity to 1-octen-3-ol with an average EC50 of 1.44 μM (Fig. 2B).

The sensitivity of the OR47a to pentyl acetate varied between different studies. In *Xenopus* oocytes, a range of 50–300 μM could trigger receptor responses^9^, while in a similar system the calculated EC50 was 10.7 ± 2.0 µM^39^ and 30.8 ± 1.28 µM^34^. In our OR47a cell line, the EC50 was 2.22 μM for pentyl acetate (Fig. 2B), which indicates about five times higher sensitivity.

For the OR49b cell line, we used styrene as a reference ligand (see "Plant disease-related VOC measurements on the OR cell lines". below) but measured the EC50 for 2-methylphenol (*o*-cresol), too. With 13.95 μM (Supplementary file Data summary.xlsx) of EC50 our OR49b cell line was 17-times more sensitive to this ligand than previously measured in *Xenopus* oocytes in which the calculated EC50 was 239 ± 76 µM^39^. In a recent study, the same OR was expressed transiently in HEK293T cells, which reacted to 2-methylphenol above the limit of detection in a 2 μM to 800 μM concentration range. Though the EC50 was not calculated, 80 μM concentration triggered a 38.5% response^40^.

The OR85b receptor response to 2-heptanone was measured previously in *Xenopus* oocytes by Nichols and Luetje^42^ with an EC50 of 70 ± 20 μM, and by Misawa et al.^41^ with an average EC50 of 45.6 μM compared to the average 68.9 μM value we obtained (Fig. 2B).

### Plant disease-related VOC measurements on the OR cell lines

Once responsive OR cell lines were selected, we tested them for sensing fungal pathogens (powdery mildew, *Botrytis cinerea*, *Fusarium* spp., *Pyrenophora* spp., and gray mold) of important crop plants (wheat, barley, grape, lettuce, rape, spinach, and strawberry). To this end, we had compiled a pathogen-related VOC catalog primarily based on our GC–MS analyses of infected samples (published results^18^ and unpublished data of our laboratory) and also systematically retrieved from published literature data^47–50^.

**Figure 3:**
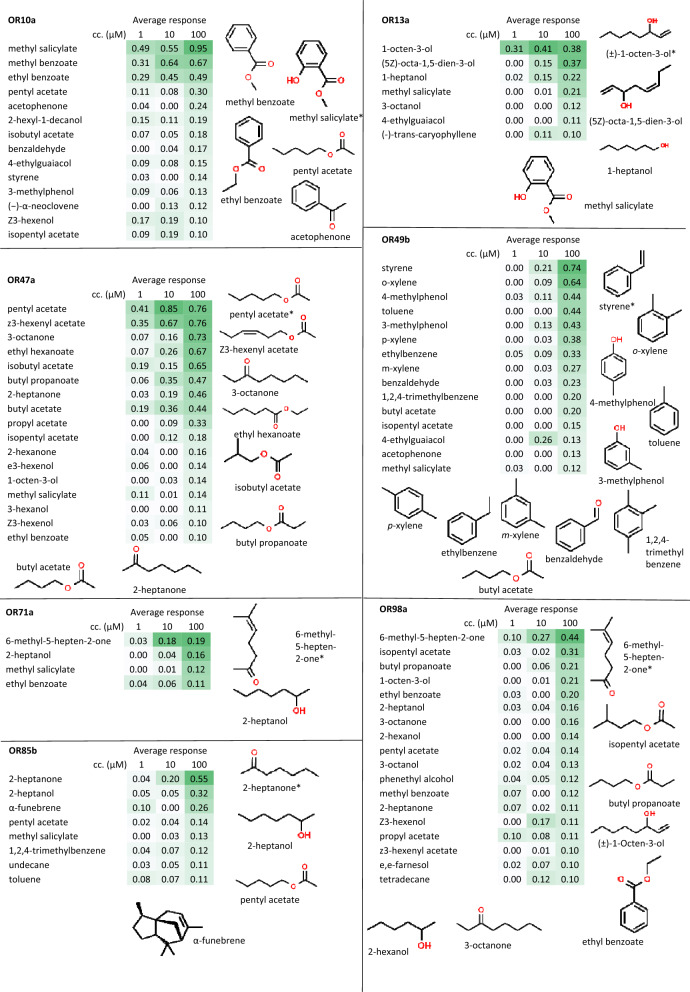
VOC ligand profile of the OR cell lines. Each VOC was measured at 1, 10, and 100 µM final concentration on each receptor. VUAA1-normalized response was calculated as described in methods. Values are averages of at least three biological replicates for responsive combinations and two for the non-responsive combinations. The chemical structures of the strong and medium ligands are shown for each cell line. Heat map: white-green colors correspond to the lowest-highest values measured for each cell line.

Together with strong ligands for the seven ORs according to the DoOR database, a set of 66 compounds from our compilation was measured on all OR cell lines in 1 μM, 10 μM, and 100 μM concentrations (Fig. 3, Supplementary Table S2).

Among the 1386 VOC concentration-OR combinations measured, 156 (11%) gave positive odorant responses. As the reference ligand (6-methyl-5-hepten-2-one) with the lowest overall VUAA1-normalized response elicited a 0.2 VUAA1-normalized response (OR71a, Fig. 2), this value was taken as the threshold to separate strong/medium reactions from low intensity ones. The majority (60.8%) of the responses were of low intensity, i.e., less than this 20% threshold of the VUAA1-normalized response. Reactions with strong (at least 40% of the VUAA1 response) or medium (20–40% of the VUAA1 response) intensity were recorded in 17% and 22.2% of all positive cases, respectively.

In 11 (2.4%) of the 462 VOC-OR combinations tested, the OR cell lines responded over the whole concentration range measured. High sensitivity to a ligand at the lowest concentration did not always guarantee the typical logistic relationship between ligand concentration and response intensity. Several odorants only activated ORs at the medium concentration, with moderate increase in response at higher concentrations. As an example, isopentyl acetate first activated the OR47a cell line at 10 μM and increasing the ligand concentration to 100 μM increased the response intensity from 12 to 18% (Supplementary Table S2). Some cell lines (and their ORs) showed a well-defined affinity for structurally similar compounds. For instance, OR49b was highly specific for aromatic compounds, as only these elicited strong or medium responses, with the exception of butyl acetate. Figure 3 summarizes the VOC ligand profiles of the OR cell lines and the chemical structures of strong and medium ligands.

We have a special focus on VOCs induced by wheat powdery mildew infections. Quantitative experimental data from consecutive years have therefore been collected in open field plots as well as from controlled inoculations of several cultivars. Six powdery mildew-specific volatile biomarkers have been identified and quantified in the headspace of healthy and diseased wheat plants^18^. Four of these biomarker VOCs were commercially available and three of them, 1-octen-3-ol, (5Z)-octa-1,5-dien-3-ol, and 3-octanone were in the detectable range of two OR cell lines in samples from diseased wheat plants (Table 1), while the concentration of 1-heptanol was below the detection limit of the OR13a cell line.

**Table 1:** Concentration range and detectability of major wheat powdery mildew biomarker VOCs by two OR cell lines.

Biomarker VOC	VOC concentration of DMSO samples (μM)		EC50	
	Min	Max	OR13a	OR47a
1-Octen-3-ol	0.30	79.24	1.44	
(5Z)-Octa-1,5-dien-3-ol	0.16	93.64	3.05	
3-Octanone	0.21	12.48		2.23

### Comparison of the OR cell line ligand profiles with the DoOr database

The DoOr database-derived response profiles, which are collections of in vivo measured datasets, served as a good starting point for the identification of candidate ORs and the prediction of their response profiles to our VOC ligands. The DoOR database sets the scale by comparing the measured data to each other, while we took the maximum response value for each OR induced by VUAA1 as 1, and compared the effect of all other ligands to this. This essentially creates a database with a similar response profile ranging from 0 to 1, but this way of calculating response magnitudes is clearly different from the data in the DoOR database, therefore response magnitude values cannot be directly compared. Nevertheless, it is an important issue to investigate how much functional responses in an expression system correspond with in vivo data.

Twenty-two out of the 66 VOCs tested in the present study have not been tested in the DoOR database, so receptor responses to these VOCs represent new information. OR10a was the best-studied receptor for our ligand set; the DoOR database contained response values for 41 VOCs, while OR71a was the least studied with only 21 previously measured VOCs. The OR10a, OR47a, and OR49b cell lines responded at 100 μM to all VOCs that were predicted as a strong ligand (at least 0.4 response) for these ORs according to the DoOR database (5/5, 8/8 and 2/2, resp. in Supplementary Table S2). Similarly, a high matching rate (11/15, 73%) was also found for the OR98a cell line. However, for three cell lines, OR13a, OR85b and OR71a the respective success rates were only 57% (4/7), 30% (3/10), and 25%(1/4). These observations indicate the importance of testing the ligand profile and sensitivity in different expression systems, and emphasize the potential role of cellular environment in determining sensitivity profiles of individual ORs.

### Concluding remarks

We generated responsive OR cell lines which stably express seven functional *Drosophila* ORs together with GCaMP6, a fluorescent calcium ion indicator protein. These cell lines were produced by transfection of HEK293 cells with a novel BAC expression vector carrying an open chromatin region to ensure higher and more stable expression levels in a long term. The OR cell lines detected odorant molecules from the ppb concentration in the liquid phase. The response was quick with the cells reaching maximum fluorescence intensity change within 10 s. Peaks of fluorescence intensity change were as high as 462% for the ORCO agonist VUAA1 and 339% for reference ligands, which is outstanding compared to the published data of ca. 50%. The concentration–response profiles of the OR cell lines were mapped for 66 plant- and plant pathogen-derived VOCs over a concentration range of three orders of magnitude. We recorded ligand-specific fluorescence responses in 11% of all 1386 measured combinations of VOC concentrations and OR cell lines, similarly to the 17% described by Hallem et al.^17^. In terms of plant pathogen-related VOC sensing, three major volatile biomarkers specific for powdery mildew infection were in detectable concentration in diseased wheat samples^18^ for the OR13a and OR47a cell lines. The OR proteins expressed here can be purified from the cells and coupled to other signal-transducing elements. For instance, systems like the ones developed earlier^37,51^ may be utilized for our assay too, and such coupling steps could further improve sensitivity for the ligands explored here.

## Materials and methods

### Molecular cloning of OR genes into plasmid and BAC expression vectors

The OR genes OR7a (FlyBase ID FBtr0071186), OR47a (FBtr0088111), and a codon-optimized version of ORCO (FBtr0113193) were synthesized (Thermo Fisher Scientific) as double-stranded linear gene fragments. The cDNAs of the OR10a, OR13a, OR19a, OR47b, OR49b, OR67b, OR69a, OR71a, OR85b, OR98a genes were cloned from the wild-type Canton S strain of *D. melanogaster*. Total RNA was extracted from adult flies by TRIzol Reagent (Thermo Fisher Scientific) according to the manufacturer’s instructions. Total RNA (1 μg) was treated with DNase I, then reverse transcribed with oligo(dT)_12–18_ primers and SuperScript IV enzyme (Invitrogen). The OR genes were amplified with gene-specific primers containing an *Asc*I recognition sequence on both primers (except *Swa*I for OR19a) and a Kozak consensus sequence on the forward primers (Supplementary Table S1).

The above 12 OR genes linked to a wild-type internal ribosome entry site from the encephalomyocarditis virus (EMCV *I*RES) and the *m*Cherry fluorescent protein gene^52^ were inserted into a modified pBlueScript KS (Agilent/Stratagene) called plasmid “L” ^22^. The OR genes were inserted into this vector at the *Asc*I (*Swa*I) restriction site, while IRES-mCherry at the *Pme*I restriction site and the resulting *p*lasmids were named as MIO(receptor name)p. The N-terminus of *O*RCO was fused to *G*CaMP6^21^ via a *G*ly_2_SerGly_3_SerGly linker. Subsequently, this fusion gene was cloned into a modified plasmid “L” at the *Asc*I restriction site, in which the CAG promoter^24^ was replaced by a *D*oxycycline-inducible Ptet-T6 bidirectional promoter^26^ and its cognate transactivator protein (3G) gene (derived from AAVS1_Puro_Tet3G_3xFLAG_Twin_Strep, a gift from Yannick Doyon: Addgene plasmid #92099^53^. The resulting plasmid was named as DOGGp in this work. MIO13a/47a/85b/98aDOGGp plasmids were generated by ligating *Sal*I- and *Srf*I-digested MIOp with *Bsu*36I-digested DOGGp (Fig. 1A). All enzymes were purchased from Thermo Fisher Scientific (except for *Srf*I, which was from New England Biolabs). Cloning was performed in the *E. coli* DH10B strain. All plasmid sequences are available upon request.

For the generation of the BAC expression vectors, a modified version of the murine *Rosa26* BAC DNA (BAC PAC Resources Children’s Hospital Oakland Research Institute clone number: RP24–85I15) was used as the vector backbone. Briefly, the original 210-kb BAC was shortened by removing approximately 70–70 kb DNA sequences from both ends of the genomic insert. First, two targeting constructs carrying 50 bp long homologous sequence regions (HR) on both ends (5′ targeting construct: HR–frt–Kanamycin–frt–HR, 3′ targeting constructs: HR–frt3–Ampicillin–frt3–HR) were generated by PCR (primers in Supplementary Table S1). The 5′ targeting construct was recombined into the targeted region replacing the original sequence by ET-cloning/recombineering^54,55^, then the cassette was excised by FLPe recombinase^56^. Subsequently, recombination and cassette excision were repeated with the 3′ targeting construct.

For ET-cloning DH10B *E. coli* harboring the original 210-kb BAC^Rosa26^ were electroporated with the temperature sensitive pSC101-BAD-gbaA (pRed/ET plasmid on Supplementary Fig. 4) plasmid which carries the recombinase proteins necessary for homologous recombination. Cells harboring the pSC101-BAD-gbaA plasmid were selected in tetracycline (5 μg/ml) at 30 °C overnight. Bacterial cells derived from one single positive colony were cultured overnight at 30 °C and transferred to 50 ml of fresh medium next day. At an optical density (OD600) of 0.2, the expression of the recombinogenic proteins was induced by the addition of l-arabinose (to a 0.3–0.4% final concentration) and by shifting the temperature to 37 °C. After one additional hour, cells were harvested and electrocompetent cells were prepared by a double wash with ice cold distilled water. Cells were resuspended in 10% ice cold glycerol solution and aliquoted to Eppendorf tubes (100 μl) prior snap freezing in liquid N_2_ or directly used for electroporation. 100 ng of linear DNA was electroporated to these cells with a Gene Pulser Xcell (BioRad) electroporator. Cells were incubated for 70 min at 37 °C without antibiotics in LB medium and plated on LB-agar containing the respective antibiotic (15 μg/ml kanamycin after 5′ targeting construct recombination and 100 μg/ml ampicillin after 3′ targeting construct recombination) at 37 °C overnight.

In the next step, bacterial cells derived from one single positive colony were cultured overnight at 37 °C in LB/antibiotic (ampicillin/kanamycin). The next day electrocompetent cells were prepared the same way as for ET-cloning and were electroporated with the expression plasmid 706 FLP which expresses the FLPe recombinase^56^ to excise the antibiotic cassette flanked by frt/frt3 sites in the 5′/3′ targeting constructs. Cells were resuspended in LB medium without antibiotics after electroporation and incubated for 70 min at 30 °C. 100 μl of cell suspension was plated into LB-agar containing tetracycline (3 μg/ml) and the appropriate antibiotics for the BAC (chloramphenicol 12.5 µg/ml) and incubated overnight at 30 °C. The plate was incubated at 37 °C for 4–6 h. 10 single colonies were picked and streaked out to another LB agar/chloramphenicol plate from the left to the right, each in one row and cultured 37 °C overnight. Single colonies were picked from this plate and we plate them on replica plates either containing only chloramphenicol or chloramphenicol and the antibiotic derived from 5′/3′ targeting constructs (ampicillin/kanamycin). Some of the colonies will not grow on ampicillin/kanamycin plates anymore, here the recombination was successful.

After recombination and cassette excision was complete with the 5′ targeting construct it was repeated the same way with the 3′ targeting construct (see experimental outline in Supplementary Fig. 4).

DOGGp and MIO13a/47a/85b/98aDOGGp plasmids were linearized by *Sfa*AI and *Pac*I restriction enzymes to generate the linear fragments required for BAC recombination into the 2nd exon of the *Rosa26* gene by ET-cloning/Recombineering as described in detail^22^. The obtained BAC vectors were designated as DOGGB and MIO13a/47a/85b/98aDOGGB, respectively (Fig. 1A).

### Cell culture and stable cell line generation

HEK293 cells (ATCC CRL-1573™) were cultured in growth medium (Dulbecco’s Modified Eagle Medium with high glucose, l-glutamine, and sodium pyruvate supplemented with 10% tetracycline-free Fetal Bovine Serum from Biosera and penicillin–streptomycin mixture). Cells were kept at 5% CO_2_ and 37 °C in a humidified incubator and were passaged two–three times a week. Transfection-grade BAC DNA was purified with the NucleoBond Xtra BAC DNA purification kit (Macherey–Nagel) and was stored at 4 °C. Plasmid DNA was purified with Genopure Plasmid Midi Kit (Roche). In all cases, a total amount of 9.375 µg DNA was transfected into HEK293 cells reaching 60–70% confluency, in T-25 flasks with FuGene HD transfection reagent (Promega) at a 1:3 DNA:FuGene HD ratio. G418 antibiotic (Thermo Fisher Scientific) was added to the cells 48 h after transfection at 100 μg/mL concentration. For stable cell pool generation, cells were cultured for two weeks, then the antibiotic concentration was increased to 150 μg/mL and cells were cultured for one more week or until they divided vigorously without any visible sign of cell death.

Actively growing transfected cell pools were trypsinized and resuspended in 1 mL growth medium for FACS sorting. The OR13a/47a/85b/98a cell lines were sorted for mCherry fluorescence without doxycycline addition, while the OR10a/49b/71a cell lines were sorted 48 h after doxycycline addition for GFP and mCherry fluorescence with a FACSAria III cell sorter (BD Biosciences) into 96-well cell culture microplates. Clonal cell lines were cultured in growth medium supplemented with 150 μg/mL G418 and observed under an Eclipse Ti2 inverted fluorescence microscope (Nikon) when starting to grow in the 96-well microplates. Four to six actively growing mCherry positive cell clones were selected and expanded for testing their VOC response. Odorant-responsive clonal cell lines were selected and cryopreserved for subsequent experiments.

### Calcium ion fluorescence assay

Twelve thousand to 18,000 cells were plated into each well of a black wall, transparent bottom 384-well microplate (Greiner) 48 h or 72 h before the measurements and were simultaneously treated with 1 μg/mL doxycycline. Typically, three columns were filled with one OR expressing cell line, each row was treated with the same compound (three technical replicates for each OR), and each responsive VOC-OR combination was measured at least three times on different days. The plate has 24 columns, thus we could record responses in up to eight different cell lines in each plate. On the day of the experiment, the growth medium was replaced with 20 µL assay buffer/well (140 mM NaCl, 5 mM KCl, 2 mM CaCl_2_, 1 mM MgCl_2_, 5 mM HEPES-Na, 20 mM glucose, pH 7.3, 340 mOsm). The fluorescence was excited at 485 nm and the emission was recorded at 535 nm with a SpectraMax iD3 multimode microplate reader (Molecular Devices) mounted with a two-channel injector. Baseline fluorescence (F0) was recorded for 5 s, after which 7 µL VOC buffer (140 mM NaCl, 5 mM KCl, 2 mM CaCl_2_, 1 mM MgCl_2_, 5 mM HEPES-Na, 31 mM glucose, 0.1% DMSO, pH 7.3, 340 mOsm) was injected into each well in one row and the fluorescence response was recorded for an additional 30 s, at a rate of one read/sec. VOC solutions (VOC buffer with 4× concentration of the final measured VOC concentration, 9 µL/well was injected) were measured on the same wells in a second round with the same protocol. Each individual concentration of a specific VOC sample was recorded in three wells for the same cell line on the same microplate. The raw data were exported in xls file format, F0 and FMAX values, and the fluorescence intensity changes (∆F) were calculated in Excel (Microsoft) (Supplementary file Data summary.xlsx) using the following equation: $\Delta \mathrm{F}=\frac{FMAX-F0}{F0}$ (see also Fig. 1C). On each plate, we recorded the response of each cell line to 50 µM VUAA1. The average of these ∆F values of each cell line were used to calculate the VUAA1-normalized values for the rest of the plate, by dividing VOC response ∆F values by the average VUAA1 response ∆F value of the corresponding OR cell line. Each VOC-OR combination was measured three times as technical replicates. The outliers were removed based on the suggestion of Grubbs outlier test. The average of these replicates were used as one ∆F and VUAA1-normalized ∆F in the following analysis.

In the next rows we tested the reference VOCs (or other VOCs known to activate the corresponding cell lines), in order to test if all cell lines are responsive. The remaining rows were used to test VOCs with unknown effect.

The recorded FMAX, F0, ∆F, VUAA1-normalized ∆F values, as well as the OR name, the name of the tested sample, and the final concentration for each well were copied into supplementary file Data summary.xlsx, that was used to summarize all the plates recorded. Here, the averages of the measurements were calculated for each receptor-VOC-concentration combination. Responses where the VUAA1-normalized ∆F values did not exceed 0.05 were considered as 0.

### Chemicals and chemical dilutions

All VOCs were purchased from Sigma-Aldrich (except for (5Z)-octa-1,5-dien-3-ol from Toronto Research Chemicals) in the highest available purity. VOCs were diluted either directly in the VOC dilution buffer or first in DMSO for water-insoluble compounds.

### Supplementary Information

Supplementary Figures.

Supplementary Table 1.

Supplementary Table 2.

Supplementary Information.

## Data Availability

The datasets generated in the current study are available in our local computers. Access to the datasets can be granted upon reasonable request. Researchers interested in accessing the data may contact the corresponding author for inquiries and requests.
