# Supplementary material for: High-throughput ligand profile characterization in novel cell lines expressing seven heterologous insect olfactory receptors for the detection of volatile plant biomarkers
Source: Sci Rep. 2023 Dec 8;13:21757. doi: 10.1038/s41598-023-47455-4 (PMC10709440; doi:10.1038/s41598-023-47455-4)
Supplement: Supplementary file 1 — Supplementary Figures. [file 41598_2023_47455_MOESM1_ESM.pptx]

## Slide 1
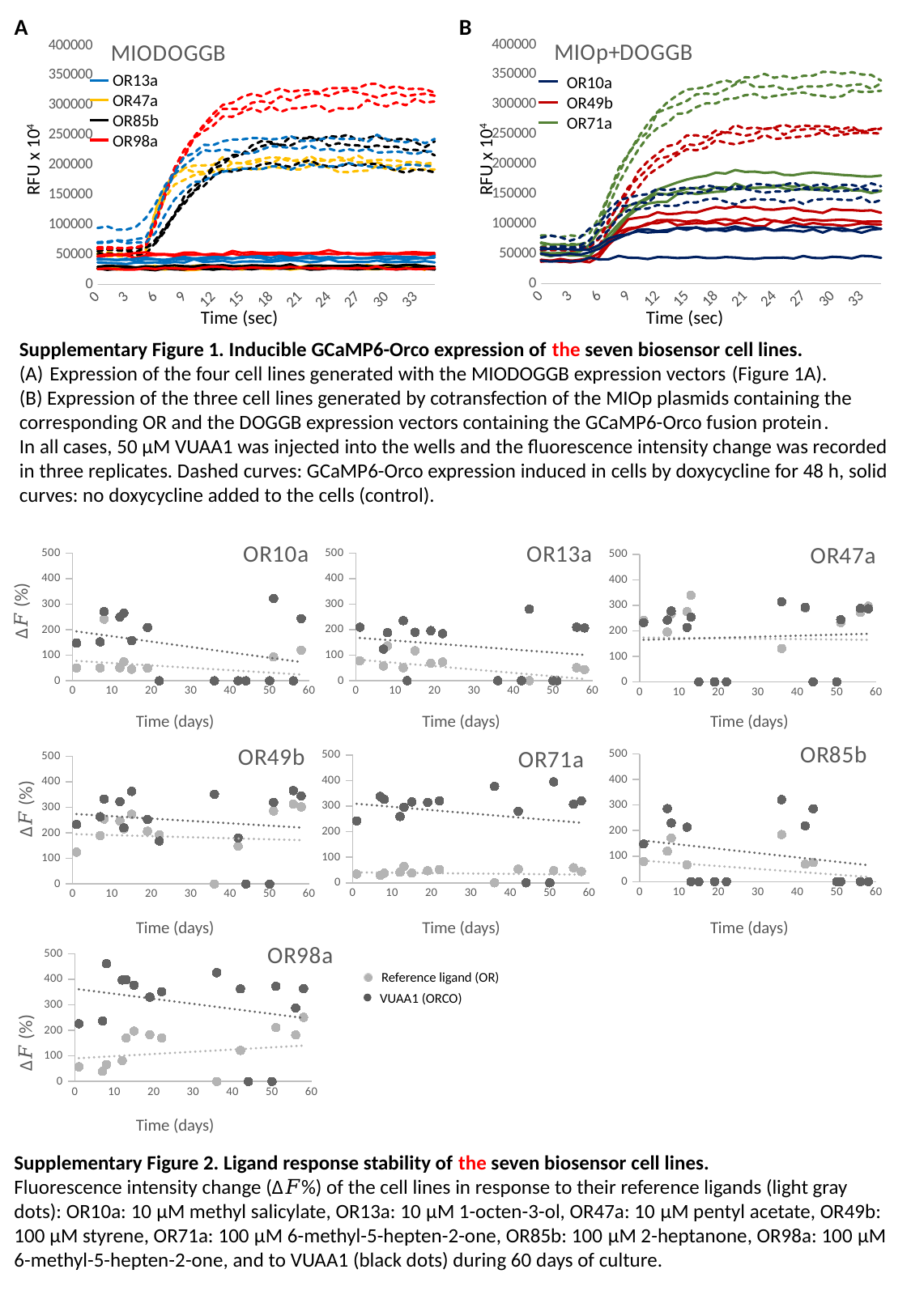

A
B
### Chart: MIOp+DOGGB
| Category | | | | | | | | | | | | | | | | | | |
|---|---|---|---|---|---|---|---|---|---|---|---|---|---|---|---|---|---|---|
| 0 | 67731.0 | 80547.0 | 61653.0 | 53035.0 | 69007.0 | 49383.0 | 56183.0 | 60529.0 | 57011.0 | 39214.0 | 40080.0 | 36798.0 | 60264.0 | 57711.0 | 76930.0 | 38598.0 | 50145.0 | 57470.0 |
| 1 | 63967.0 | 80636.0 | 64720.0 | 50755.0 | 65585.0 | 45904.0 | 57710.0 | 60445.0 | 55937.0 | 38328.0 | 38228.0 | 38264.0 | 60077.0 | 61470.0 | 80411.0 | 36988.0 | 47600.0 | 58556.0 |
| 2 | 65564.0 | 77514.0 | 65865.0 | 51371.0 | 65491.0 | 49562.0 | 57089.0 | 61313.0 | 56248.0 | 36655.0 | 41413.0 | 38210.0 | 63131.0 | 60854.0 | 77804.0 | 38237.0 | 47636.0 | 57922.0 |
| 3 | 65191.0 | 80902.0 | 66383.0 | 52111.0 | 64342.0 | 47318.0 | 55348.0 | 57960.0 | 55989.0 | 37881.0 | 38756.0 | 35629.0 | 61223.0 | 59164.0 | 71982.0 | 37462.0 | 49993.0 | 57644.0 |
| 4 | 66209.0 | 79291.0 | 65465.0 | 53455.0 | 65994.0 | 47230.0 | 56912.0 | 61488.0 | 57731.0 | 40145.0 | 37755.0 | 38003.0 | 59757.0 | 63820.0 | 76946.0 | 37897.0 | 48318.0 | 56692.0 |
| 5 | 70145.0 | 84041.0 | 68712.0 | 65260.0 | 57442.0 | 45965.0 | 57107.0 | 60535.0 | 60265.0 | 42407.0 | 42041.0 | 35895.0 | 64336.0 | 72657.0 | 92162.0 | 43564.0 | 52709.0 | 57211.0 |
| 6 | 100220.0 | 111273.0 | 97654.0 | 88646.0 | 64077.0 | 63786.0 | 64279.0 | 75222.0 | 77778.0 | 50573.0 | 51164.0 | 45088.0 | 77195.0 | 91754.0 | 100930.0 | 40563.0 | 58506.0 | 64760.0 |
| 7 | 147539.0 | 149931.0 | 131326.0 | 95778.0 | 82172.0 | 74595.0 | 91142.0 | 103754.0 | 106324.0 | 70759.0 | 71816.0 | 65538.0 | 90045.0 | 114914.0 | 123346.0 | 41005.0 | 69520.0 | 75750.0 |
| 8 | 183855.0 | 193036.0 | 167799.0 | 114687.0 | 100271.0 | 94162.0 | 114443.0 | 134196.0 | 131148.0 | 88723.0 | 86710.0 | 80564.0 | 111859.0 | 129381.0 | 139479.0 | 46512.0 | 76299.0 | 80874.0 |
| 9 | 216939.0 | 217644.0 | 197360.0 | 130382.0 | 115971.0 | 113307.0 | 141730.0 | 161445.0 | 155055.0 | 107865.0 | 92683.0 | 89931.0 | 123118.0 | 130823.0 | 144118.0 | 43502.0 | 83577.0 | 82239.0 |
| 10 | 240947.0 | 244302.0 | 223843.0 | 135599.0 | 133249.0 | 125665.0 | 161071.0 | 176821.0 | 187527.0 | 110425.0 | 94248.0 | 94629.0 | 124967.0 | 136381.0 | 147701.0 | 43161.0 | 87783.0 | 90202.0 |
| 11 | 253380.0 | 267473.0 | 246158.0 | 144502.0 | 141978.0 | 134057.0 | 181878.0 | 192164.0 | 204796.0 | 112433.0 | 100955.0 | 98819.0 | 125784.0 | 135315.0 | 149934.0 | 41202.0 | 85629.0 | 90936.0 |
| 12 | 286660.0 | 287473.0 | 260473.0 | 157454.0 | 151025.0 | 136539.0 | 193322.0 | 208832.0 | 217628.0 | 119480.0 | 101208.0 | 106211.0 | 131677.0 | 149614.0 | 158902.0 | 44482.0 | 86731.0 | 88017.0 |
| 13 | 293008.0 | 303364.0 | 268820.0 | 163372.0 | 149540.0 | 137240.0 | 203096.0 | 212595.0 | 230045.0 | 116379.0 | 105180.0 | 106634.0 | 130104.0 | 148254.0 | 156134.0 | 44159.0 | 86896.0 | 89881.0 |
| 14 | 299000.0 | 315093.0 | 284163.0 | 169229.0 | 154025.0 | 149359.0 | 215521.0 | 219770.0 | 233930.0 | 117121.0 | 97753.0 | 106111.0 | 130628.0 | 150517.0 | 158971.0 | 42936.0 | 93079.0 | 93046.0 |
| 15 | 305840.0 | 324981.0 | 285549.0 | 174231.0 | 158834.0 | 152784.0 | 224882.0 | 233314.0 | 248127.0 | 119962.0 | 100269.0 | 103676.0 | 136295.0 | 150538.0 | 161055.0 | 43429.0 | 90046.0 | 93440.0 |
| 16 | 317035.0 | 326632.0 | 295467.0 | 178095.0 | 160411.0 | 150067.0 | 223458.0 | 231706.0 | 248411.0 | 119119.0 | 101309.0 | 106296.0 | 135931.0 | 152956.0 | 158660.0 | 44899.0 | 94495.0 | 93841.0 |
| 17 | 318182.0 | 329260.0 | 301921.0 | 180484.0 | 156193.0 | 152030.0 | 227523.0 | 238347.0 | 245109.0 | 124073.0 | 100816.0 | 102933.0 | 140982.0 | 153525.0 | 164653.0 | 43002.0 | 88670.0 | 91347.0 |
| 18 | 317102.0 | 335651.0 | 301706.0 | 185946.0 | 161492.0 | 153768.0 | 240434.0 | 247941.0 | 256244.0 | 128164.0 | 102730.0 | 106821.0 | 142521.0 | 160251.0 | 161898.0 | 42798.0 | 90471.0 | 91555.0 |
| 19 | 324943.0 | 340278.0 | 308969.0 | 184344.0 | 161383.0 | 154649.0 | 235923.0 | 253671.0 | 252530.0 | 125976.0 | 98205.0 | 107982.0 | 137280.0 | 157386.0 | 157292.0 | 44625.0 | 88036.0 | 92583.0 |
| 20 | 333631.0 | 335983.0 | 310209.0 | 190323.0 | 157759.0 | 167522.0 | 233233.0 | 251494.0 | 265861.0 | 129445.0 | 101967.0 | 105018.0 | 132736.0 | 158535.0 | 163759.0 | 43806.0 | 91951.0 | 95853.0 |
| 21 | 338363.0 | 345483.0 | 315433.0 | 187215.0 | 159727.0 | 161600.0 | 237474.0 | 247644.0 | 259174.0 | 127071.0 | 100713.0 | 110006.0 | 139581.0 | 158600.0 | 160971.0 | 42219.0 | 88395.0 | 91726.0 |
| 22 | 329250.0 | 345167.0 | 317680.0 | 187703.0 | 160079.0 | 160363.0 | 239939.0 | 250712.0 | 261778.0 | 128216.0 | 96481.0 | 104981.0 | 141686.0 | 155316.0 | 167243.0 | 41536.0 | 89960.0 | 93438.0 |
| 23 | 329316.0 | 351052.0 | 310943.0 | 185494.0 | 161238.0 | 161353.0 | 249456.0 | 259139.0 | 258351.0 | 124188.0 | 95879.0 | 108141.0 | 140494.0 | 152210.0 | 166084.0 | 43150.0 | 91999.0 | 93623.0 |
| 24 | 331613.0 | 344723.0 | 314243.0 | 186638.0 | 164171.0 | 161058.0 | 242212.0 | 261135.0 | 261545.0 | 123877.0 | 106041.0 | 104934.0 | 140773.0 | 154212.0 | 163551.0 | 43206.0 | 88846.0 | 89080.0 |
| 25 | 325319.0 | 347558.0 | 316373.0 | 182246.0 | 159470.0 | 165370.0 | 243581.0 | 249358.0 | 266057.0 | 126050.0 | 102008.0 | 103386.0 | 136333.0 | 154224.0 | 165072.0 | 43831.0 | 89748.0 | 93786.0 |
| 26 | 325753.0 | 343975.0 | 318662.0 | 181760.0 | 157249.0 | 162108.0 | 245114.0 | 256913.0 | 261465.0 | 126997.0 | 102544.0 | 109584.0 | 140757.0 | 156627.0 | 169627.0 | 42112.0 | 88999.0 | 97452.0 |
| 27 | 330402.0 | 347861.0 | 320029.0 | 184576.0 | 158165.0 | 165437.0 | 246645.0 | 258095.0 | 264974.0 | 122243.0 | 98091.0 | 105836.0 | 133569.0 | 159397.0 | 159570.0 | 46378.0 | 87104.0 | 94889.0 |
| 28 | 329991.0 | 348205.0 | 315908.0 | 186235.0 | 157605.0 | 159801.0 | 248034.0 | 259787.0 | 263412.0 | 121078.0 | 101659.0 | 107252.0 | 140412.0 | 156993.0 | 163790.0 | 43137.0 | 91034.0 | 94089.0 |
| 29 | 330332.0 | 354570.0 | 314351.0 | 184797.0 | 156255.0 | 161036.0 | 254051.0 | 256009.0 | 263710.0 | 118444.0 | 99237.0 | 105425.0 | 141432.0 | 158144.0 | 167024.0 | 42706.0 | 85515.0 | 94414.0 |
| 30 | 333712.0 | 354091.0 | 311652.0 | 183618.0 | 157741.0 | 159181.0 | 247596.0 | 259063.0 | 262141.0 | 125039.0 | 98824.0 | 106491.0 | 141819.0 | 154196.0 | 161201.0 | 45075.0 | 91802.0 | 92523.0 |
| 31 | 329771.0 | 351471.0 | 314089.0 | 182085.0 | 161518.0 | 160556.0 | 249258.0 | 253356.0 | 258376.0 | 123228.0 | 96771.0 | 107040.0 | 140393.0 | 154855.0 | 165359.0 | 44302.0 | 88385.0 | 93074.0 |
| 32 | 332684.0 | 354000.0 | 323814.0 | 181229.0 | 156123.0 | 156314.0 | 251748.0 | 257310.0 | 263159.0 | 123147.0 | 98302.0 | 105691.0 | 139926.0 | 156440.0 | 165178.0 | 42316.0 | 89260.0 | 88755.0 |
| 33 | 333804.0 | 350307.0 | 320953.0 | 179668.0 | 155694.0 | 155949.0 | 252972.0 | 255962.0 | 252457.0 | 121993.0 | 101245.0 | 103862.0 | 144897.0 | 160242.0 | 161877.0 | 46651.0 | 85277.0 | 93778.0 |
| 34 | 326230.0 | 349395.0 | 321424.0 | 179331.0 | 151909.0 | 152002.0 | 252133.0 | 257626.0 | 258337.0 | 122799.0 | 99511.0 | 104055.0 | 134931.0 | 155308.0 | 167886.0 | 45863.0 | 90270.0 | 95432.0 |
| 35 | 335346.0 | 339934.0 | 323040.0 | 181090.0 | 155076.0 | 155750.0 | 251287.0 | 259348.0 | 260656.0 | 118675.0 | 99004.0 | 104021.0 | 139988.0 | 156379.0 | 162980.0 | 43303.0 | 91936.0 | 91377.0 |
### Chart: MIODOGGB
| Category | | | | | | | | | | | | | | | | | | | | | | | | |
|---|---|---|---|---|---|---|---|---|---|---|---|---|---|---|---|---|---|---|---|---|---|---|---|---|
| 0 | 50804.0 | 52335.0 | 48043.0 | 26338.0 | 29492.0 | 25900.0 | 55363.0 | 50960.0 | 57957.0 | 29605.0 | 27165.0 | 26503.0 | 62342.0 | 59720.0 | 60879.0 | 45840.0 | 47821.0 | 25973.0 | 94611.0 | 69499.0 | 70854.0 | 36912.0 | 43354.0 | 41415.0 |
| 1 | 54076.0 | 52022.0 | 47371.0 | 27239.0 | 29223.0 | 27404.0 | 58135.0 | 51877.0 | 60860.0 | 31507.0 | 24592.0 | 26549.0 | 62773.0 | 59825.0 | 59913.0 | 48356.0 | 50798.0 | 26746.0 | 97105.0 | 69845.0 | 71623.0 | 36178.0 | 42003.0 | 42026.0 |
| 2 | 51221.0 | 51271.0 | 47584.0 | 27803.0 | 28504.0 | 25298.0 | 57146.0 | 50830.0 | 59915.0 | 30632.0 | 25860.0 | 25495.0 | 61289.0 | 61039.0 | 60562.0 | 49666.0 | 49770.0 | 25717.0 | 91680.0 | 73430.0 | 74037.0 | 35596.0 | 40983.0 | 41024.0 |
| 3 | 52376.0 | 51276.0 | 47212.0 | 25490.0 | 28178.0 | 25566.0 | 56095.0 | 49080.0 | 57813.0 | 30600.0 | 26844.0 | 27765.0 | 60961.0 | 60531.0 | 60484.0 | 48838.0 | 51016.0 | 24810.0 | 91905.0 | 68951.0 | 72740.0 | 34310.0 | 43226.0 | 41181.0 |
| 4 | 50941.0 | 53650.0 | 47954.0 | 27423.0 | 28463.0 | 26285.0 | 54439.0 | 49198.0 | 61085.0 | 28364.0 | 26262.0 | 26683.0 | 62671.0 | 62122.0 | 61641.0 | 44152.0 | 52657.0 | 25159.0 | 96546.0 | 70539.0 | 76139.0 | 36906.0 | 38556.0 | 43060.0 |
| 5 | 59035.0 | 60436.0 | 53958.0 | 28235.0 | 30229.0 | 25516.0 | 61711.0 | 50918.0 | 61698.0 | 30539.0 | 25301.0 | 27349.0 | 63848.0 | 65426.0 | 67915.0 | 48747.0 | 53763.0 | 27346.0 | 111150.0 | 69838.0 | 78065.0 | 36221.0 | 42156.0 | 45249.0 |
| 6 | 109361.0 | 124353.0 | 120697.0 | 29725.0 | 31001.0 | 26036.0 | 68611.0 | 69781.0 | 74508.0 | 29252.0 | 23875.0 | 27135.0 | 99972.0 | 103090.0 | 105254.0 | 49430.0 | 50865.0 | 25935.0 | 134297.0 | 86180.0 | 110600.0 | 37159.0 | 42438.0 | 41921.0 |
| 7 | 144621.0 | 163234.0 | 164650.0 | 29559.0 | 28686.0 | 25084.0 | 89008.0 | 92369.0 | 96676.0 | 31079.0 | 27071.0 | 26974.0 | 147352.0 | 154368.0 | 153217.0 | 50043.0 | 50861.0 | 27911.0 | 165260.0 | 116281.0 | 153658.0 | 35607.0 | 38794.0 | 41363.0 |
| 8 | 167213.0 | 185345.0 | 181045.0 | 30146.0 | 29079.0 | 27005.0 | 116221.0 | 119400.0 | 115242.0 | 31931.0 | 26111.0 | 25341.0 | 190150.0 | 195548.0 | 192151.0 | 47285.0 | 49717.0 | 26999.0 | 188430.0 | 142386.0 | 185461.0 | 37884.0 | 42447.0 | 43615.0 |
| 9 | 174942.0 | 194949.0 | 193989.0 | 29423.0 | 27872.0 | 24016.0 | 134901.0 | 138931.0 | 147432.0 | 30760.0 | 26573.0 | 24921.0 | 213593.0 | 225191.0 | 216485.0 | 49965.0 | 53359.0 | 26274.0 | 213618.0 | 157992.0 | 199113.0 | 36641.0 | 42076.0 | 46274.0 |
| 10 | 182927.0 | 199134.0 | 188220.0 | 28588.0 | 31342.0 | 24209.0 | 154969.0 | 157097.0 | 164217.0 | 30167.0 | 26000.0 | 28189.0 | 244788.0 | 247045.0 | 244410.0 | 49662.0 | 53369.0 | 25855.0 | 219918.0 | 171579.0 | 211713.0 | 42229.0 | 43804.0 | 43215.0 |
| 11 | 185078.0 | 199407.0 | 188530.0 | 32279.0 | 32502.0 | 25979.0 | 172635.0 | 164415.0 | 176014.0 | 32361.0 | 28018.0 | 25965.0 | 263592.0 | 266802.0 | 256541.0 | 50445.0 | 49817.0 | 28337.0 | 233823.0 | 172786.0 | 225165.0 | 37825.0 | 41730.0 | 45004.0 |
| 12 | 188757.0 | 200084.0 | 192567.0 | 30921.0 | 30178.0 | 26222.0 | 194118.0 | 180614.0 | 198177.0 | 31521.0 | 24800.0 | 25232.0 | 277706.0 | 285446.0 | 266053.0 | 47032.0 | 51449.0 | 30052.0 | 233363.0 | 182720.0 | 222717.0 | 38657.0 | 43393.0 | 45852.0 |
| 13 | 191643.0 | 213372.0 | 193266.0 | 29275.0 | 30108.0 | 25893.0 | 200413.0 | 190609.0 | 208142.0 | 32342.0 | 27531.0 | 26425.0 | 293932.0 | 300730.0 | 281271.0 | 48665.0 | 49947.0 | 29049.0 | 239848.0 | 188720.0 | 226385.0 | 39842.0 | 43956.0 | 45655.0 |
| 14 | 203661.0 | 203290.0 | 192918.0 | 28764.0 | 29276.0 | 27189.0 | 216473.0 | 188885.0 | 212620.0 | 30787.0 | 25407.0 | 25792.0 | 293992.0 | 304036.0 | 280785.0 | 51826.0 | 56227.0 | 26139.0 | 242594.0 | 191766.0 | 223614.0 | 36498.0 | 42476.0 | 43789.0 |
| 15 | 202866.0 | 203216.0 | 196035.0 | 30490.0 | 30966.0 | 25057.0 | 217809.0 | 192558.0 | 218773.0 | 30454.0 | 26889.0 | 28044.0 | 293334.0 | 313827.0 | 288121.0 | 48161.0 | 52762.0 | 27403.0 | 242882.0 | 194112.0 | 220336.0 | 38458.0 | 46541.0 | 44563.0 |
| 16 | 201770.0 | 207253.0 | 192510.0 | 30007.0 | 30451.0 | 27885.0 | 232632.0 | 194904.0 | 223608.0 | 32155.0 | 25428.0 | 25907.0 | 304056.0 | 319061.0 | 295003.0 | 50424.0 | 54071.0 | 28316.0 | 245261.0 | 193893.0 | 218999.0 | 38673.0 | 45445.0 | 46966.0 |
| 17 | 207547.0 | 210365.0 | 193577.0 | 30005.0 | 31080.0 | 26656.0 | 239899.0 | 201065.0 | 231245.0 | 31640.0 | 27596.0 | 27154.0 | 306820.0 | 315313.0 | 288687.0 | 50182.0 | 52658.0 | 27686.0 | 244896.0 | 192997.0 | 225470.0 | 38446.0 | 44416.0 | 47150.0 |
| 18 | 202055.0 | 212891.0 | 197130.0 | 28127.0 | 31283.0 | 25545.0 | 238517.0 | 203250.0 | 235699.0 | 31096.0 | 27117.0 | 28800.0 | 303584.0 | 323157.0 | 298652.0 | 50060.0 | 52843.0 | 27486.0 | 242962.0 | 197591.0 | 231637.0 | 37947.0 | 40117.0 | 44859.0 |
| 19 | 210978.0 | 211989.0 | 195427.0 | 28262.0 | 28951.0 | 28163.0 | 233137.0 | 200050.0 | 233863.0 | 30526.0 | 26678.0 | 26620.0 | 314846.0 | 328019.0 | 288987.0 | 49976.0 | 53317.0 | 27409.0 | 244097.0 | 197831.0 | 230398.0 | 39971.0 | 44205.0 | 47948.0 |
| 20 | 206298.0 | 210406.0 | 192851.0 | 28218.0 | 29420.0 | 24819.0 | 246840.0 | 196905.0 | 232516.0 | 33870.0 | 26768.0 | 29410.0 | 308021.0 | 322156.0 | 294775.0 | 48507.0 | 52829.0 | 29415.0 | 248988.0 | 191821.0 | 228916.0 | 40434.0 | 42128.0 | 44677.0 |
| 21 | 209070.0 | 204028.0 | 192723.0 | 28915.0 | 29600.0 | 26752.0 | 244606.0 | 204115.0 | 239984.0 | 28068.0 | 28342.0 | 28382.0 | 315759.0 | 325140.0 | 294993.0 | 51143.0 | 52504.0 | 28244.0 | 240689.0 | 199122.0 | 226683.0 | 39780.0 | 43950.0 | 45059.0 |
| 22 | 202233.0 | 215522.0 | 197976.0 | 32133.0 | 29224.0 | 26876.0 | 244776.0 | 207857.0 | 246952.0 | 31467.0 | 25103.0 | 26355.0 | 319062.0 | 324850.0 | 297799.0 | 51016.0 | 52330.0 | 27356.0 | 240176.0 | 201398.0 | 233043.0 | 37135.0 | 43864.0 | 48391.0 |
| 23 | 211357.0 | 206990.0 | 201041.0 | 29559.0 | 30839.0 | 27216.0 | 241191.0 | 200769.0 | 244030.0 | 31704.0 | 25257.0 | 25969.0 | 319245.0 | 321319.0 | 300120.0 | 51085.0 | 53472.0 | 26580.0 | 242765.0 | 196980.0 | 225032.0 | 38612.0 | 42561.0 | 44784.0 |
| 24 | 204885.0 | 208651.0 | 195700.0 | 28854.0 | 28526.0 | 27707.0 | 248493.0 | 197567.0 | 232157.0 | 30962.0 | 27143.0 | 26985.0 | 315769.0 | 328936.0 | 289306.0 | 51328.0 | 57465.0 | 27662.0 | 245560.0 | 197594.0 | 227421.0 | 42678.0 | 42885.0 | 45878.0 |
| 25 | 213095.0 | 208201.0 | 188467.0 | 30472.0 | 31014.0 | 28282.0 | 246983.0 | 196526.0 | 236461.0 | 31029.0 | 26721.0 | 27705.0 | 321782.0 | 328579.0 | 294886.0 | 50770.0 | 53626.0 | 27195.0 | 243661.0 | 198628.0 | 222893.0 | 37901.0 | 44471.0 | 44710.0 |
| 26 | 209360.0 | 207011.0 | 187972.0 | 30747.0 | 31143.0 | 26309.0 | 250598.0 | 202552.0 | 233483.0 | 31099.0 | 26268.0 | 28406.0 | 324904.0 | 328799.0 | 294264.0 | 53031.0 | 51098.0 | 26831.0 | 243473.0 | 203385.0 | 226516.0 | 37675.0 | 44911.0 | 45853.0 |
| 27 | 204786.0 | 216089.0 | 190123.0 | 29387.0 | 29095.0 | 25925.0 | 240916.0 | 198407.0 | 232926.0 | 32363.0 | 25380.0 | 27759.0 | 321272.0 | 328358.0 | 295103.0 | 50294.0 | 52082.0 | 27692.0 | 245030.0 | 200301.0 | 223316.0 | 38218.0 | 43675.0 | 43687.0 |
| 28 | 204664.0 | 207243.0 | 190532.0 | 31203.0 | 32540.0 | 25848.0 | 245353.0 | 201875.0 | 230033.0 | 30830.0 | 26688.0 | 28647.0 | 317817.0 | 335488.0 | 308874.0 | 50094.0 | 51597.0 | 26888.0 | 238211.0 | 198349.0 | 227078.0 | 37712.0 | 43785.0 | 44133.0 |
| 29 | 204369.0 | 208628.0 | 198588.0 | 30571.0 | 29732.0 | 25476.0 | 249049.0 | 201828.0 | 232683.0 | 30170.0 | 26203.0 | 27827.0 | 313147.0 | 335943.0 | 301369.0 | 49447.0 | 52813.0 | 30211.0 | 250635.0 | 195596.0 | 221031.0 | 40221.0 | 45001.0 | 46499.0 |
| 30 | 199507.0 | 208271.0 | 193604.0 | 29284.0 | 32634.0 | 27141.0 | 240548.0 | 196092.0 | 230165.0 | 32602.0 | 27140.0 | 26595.0 | 318062.0 | 327944.0 | 298163.0 | 51629.0 | 52432.0 | 26089.0 | 243135.0 | 197202.0 | 221449.0 | 37459.0 | 41569.0 | 46731.0 |
| 31 | 197977.0 | 207561.0 | 191193.0 | 29828.0 | 31247.0 | 27938.0 | 243421.0 | 190941.0 | 230853.0 | 31235.0 | 25346.0 | 28710.0 | 320828.0 | 332018.0 | 304708.0 | 50485.0 | 51807.0 | 25333.0 | 239900.0 | 192304.0 | 222498.0 | 37607.0 | 43999.0 | 46945.0 |
| 32 | 201959.0 | 204622.0 | 194432.0 | 28874.0 | 30458.0 | 26608.0 | 243743.0 | 186996.0 | 225494.0 | 32912.0 | 26054.0 | 27699.0 | 315128.0 | 327042.0 | 300153.0 | 51146.0 | 53647.0 | 26878.0 | 238006.0 | 200298.0 | 220849.0 | 37801.0 | 41562.0 | 45970.0 |
| 33 | 200022.0 | 205498.0 | 192477.0 | 30871.0 | 33431.0 | 28433.0 | 238148.0 | 192740.0 | 225347.0 | 32441.0 | 28890.0 | 26548.0 | 317909.0 | 327729.0 | 310758.0 | 50632.0 | 54016.0 | 25679.0 | 243042.0 | 200284.0 | 218257.0 | 37263.0 | 43517.0 | 46011.0 |
| 34 | 199736.0 | 201899.0 | 192806.0 | 30792.0 | 29739.0 | 24509.0 | 230027.0 | 191392.0 | 223531.0 | 30661.0 | 26568.0 | 26730.0 | 319261.0 | 326930.0 | 304397.0 | 49992.0 | 52928.0 | 26161.0 | 236354.0 | 198635.0 | 222966.0 | 39143.0 | 44103.0 | 43511.0 |
| 35 | 200909.0 | 204515.0 | 192671.0 | None | None | None | 239762.0 | 187770.0 | 216220.0 | 29602.0 | 25043.0 | 25981.0 | 315990.0 | 321032.0 | 306428.0 | 49711.0 | 52504.0 | 27186.0 | 243750.0 | 198189.0 | 222472.0 | 36936.0 | 45152.0 | 47937.0 |OR13aOR47a
OR85b
OR98a
OR10aOR49b
OR71a
RFU x 104
RFU x 104
Time (sec)
Time (sec)
Supplementary Figure 1. Inducible GCaMP6-Orco expression of the seven biosensor cell lines.
Expression of the four cell lines generated with the MIODOGGB expression vectors (Figure 1A).
(B) Expression of the three cell lines generated by cotransfection of the MIOp plasmids containing the corresponding OR and the DOGGB expression vectors containing the GCaMP6-Orco fusion protein.
In all cases, 50 µM VUAA1 was injected into the wells and the fluorescence intensity change was recorded in three replicates. Dashed curves: GCaMP6-Orco expression induced in cells by doxycycline for 48 h, solid curves: no doxycycline added to the cells (control).
### Chart: OR13a
| Category | | |
|---|---|---|
### Chart: OR10a
| Category | | |
|---|---|---|
### Chart: OR47a
| Category | | |
|---|---|---|∆𝐹 (%)
Time (days)
Time (days)
Time (days)
### Chart: OR85b
| Category | | |
|---|---|---|
### Chart: OR71a
| Category | | |
|---|---|---|
### Chart: OR49b
| Category | | |
|---|---|---|∆𝐹 (%)
Time (days)
Time (days)
Time (days)
### Chart: OR98a
| Category | | |
|---|---|---|Reference ligand (OR)
VUAA1 (ORCO)
∆𝐹 (%)
Time (days)
Supplementary Figure 2. Ligand response stability of the seven biosensor cell lines.
Fluorescence intensity change (∆𝐹%) of the cell lines in response to their reference ligands (light gray dots): OR10a: 10 μM methyl salicylate, OR13a: 10 μM 1-octen-3-ol, OR47a: 10 μM pentyl acetate, OR49b: 100 μM styrene, OR71a: 100 μM 6-methyl-5-hepten-2-one, OR85b: 100 μM 2-heptanone, OR98a: 100 μM 6-methyl-5-hepten-2-one, and to VUAA1 (black dots) during 60 days of culture.

## Slide 2
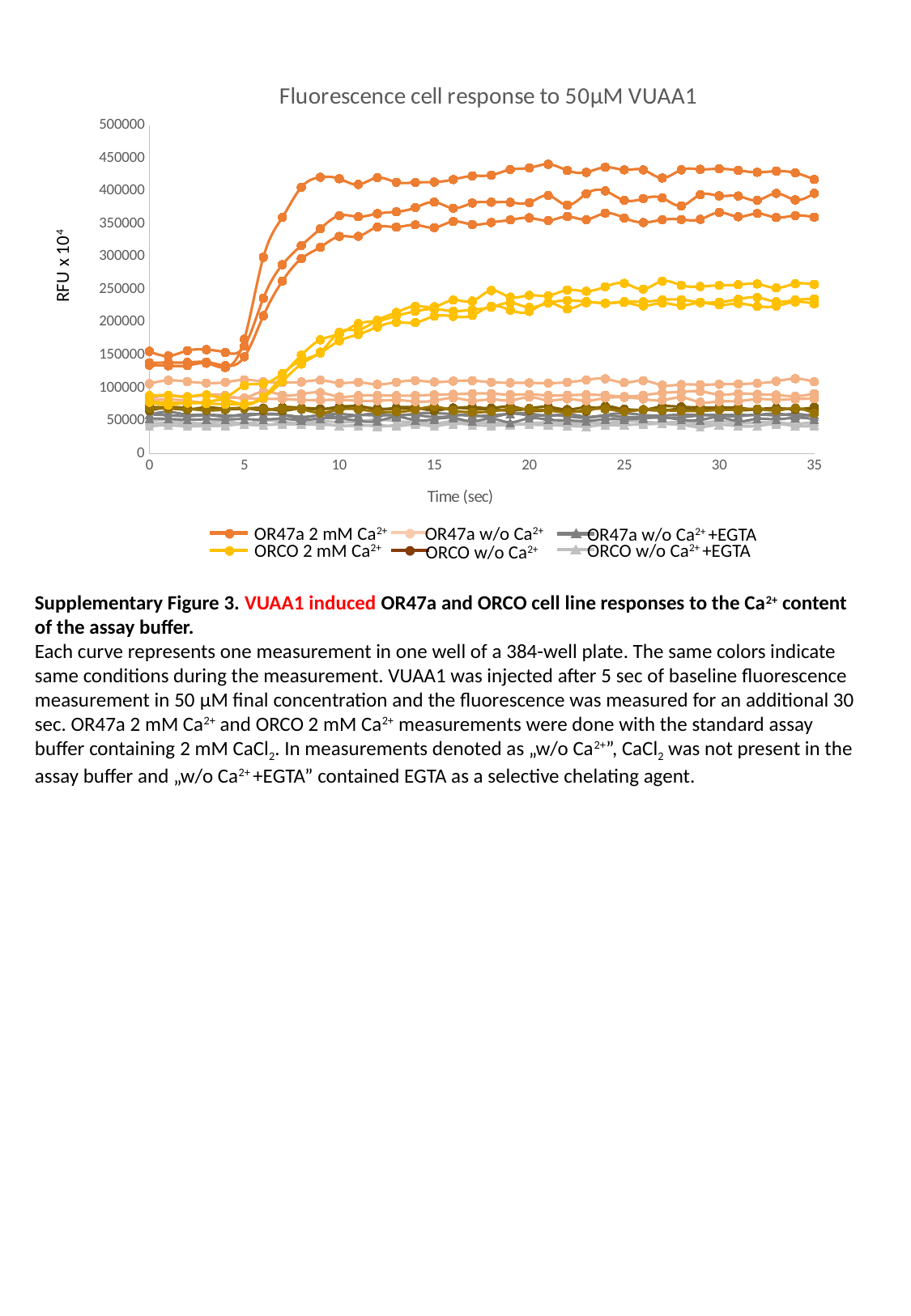

### Chart: Fluorescence cell response to 50μM VUAA1
| Category | | | | | | | | | | | | | | | | | | |
|---|---|---|---|---|---|---|---|---|---|---|---|---|---|---|---|---|---|---|OR47a 2 mM Ca2+
OR47a w/o Ca2+
OR47a w/o Ca2+ +EGTA
ORCO w/o Ca2+ +EGTA
ORCO 2 mM Ca2+
ORCO w/o Ca2+
Supplementary Figure 3. VUAA1 induced OR47a and ORCO cell line responses to the Ca2+ content of the assay buffer.
Each curve represents one measurement in one well of a 384-well plate. The same colors indicate same conditions during the measurement. VUAA1 was injected after 5 sec of baseline fluorescence measurement in 50 μM final concentration and the fluorescence was measured for an additional 30 sec. OR47a 2 mM Ca2+ and ORCO 2 mM Ca2+ measurements were done with the standard assay buffer containing 2 mM CaCl2. In measurements denoted as „w/o Ca2+”, CaCl2 was not present in the assay buffer and „w/o Ca2+ +EGTA” contained EGTA as a selective chelating agent.

## Slide 3
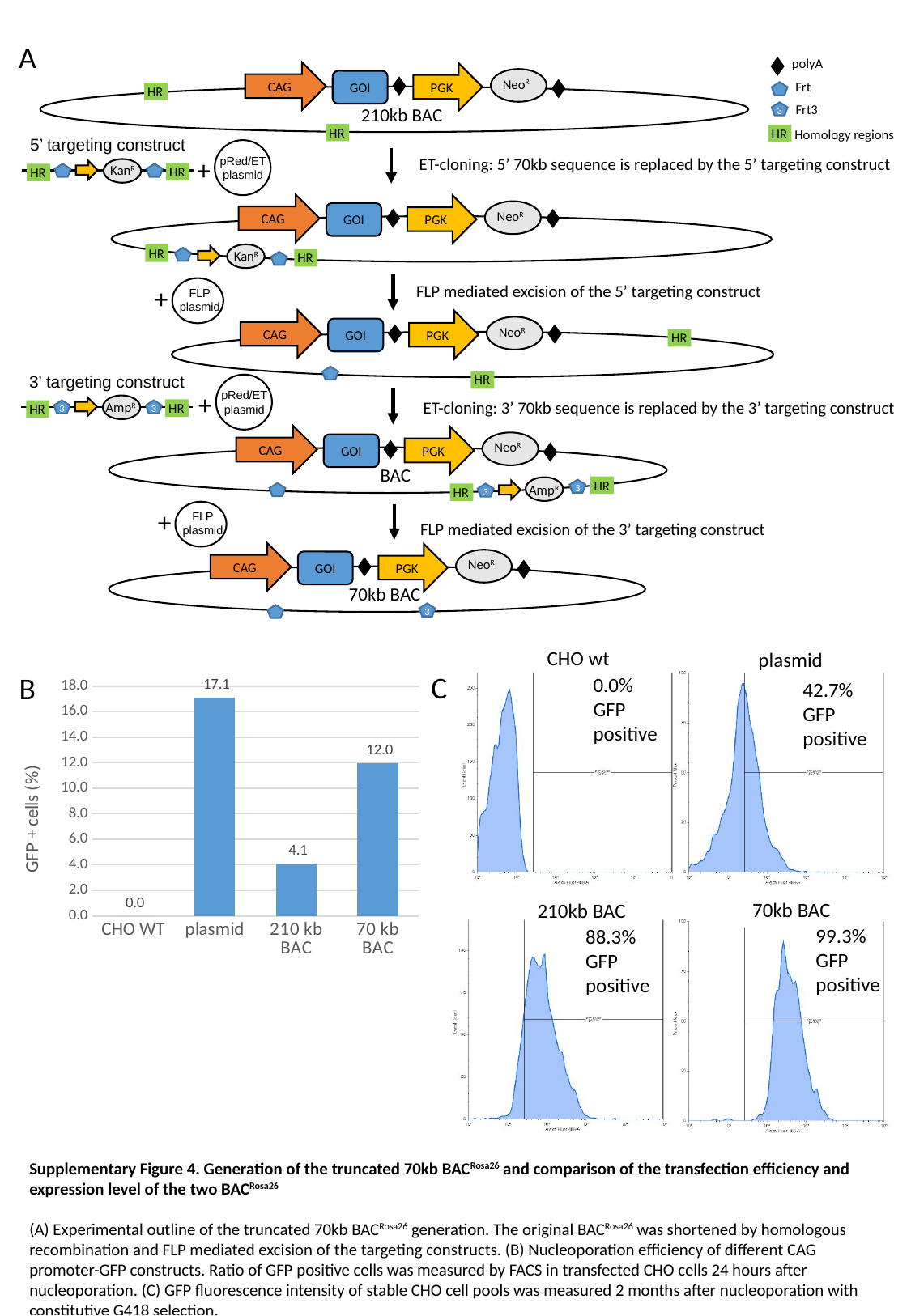

A
polyA
Frt
Frt3
3
HR
Homology regions
CAG
PGK
NeoR
GOI
HR
~210kb BAC
HR
5’ targeting construct
KanR
HR
HR
+
pRed/ET plasmid
ET-cloning: 5’ 70kb sequence is replaced by the 5’ targeting construct
CAG
PGK
NeoR
GOI
~
HR
HR
KanR
FLP mediated excision of the 5’ targeting construct
+
FLP plasmid
CAG
PGK
NeoR
GOI
HR
~
HR
3’ targeting construct
AmpR
HR
HR
3
3
+
pRed/ET plasmid
ET-cloning: 3’ 70kb sequence is replaced by the 3’ targeting construct
CAG
PGK
NeoR
GOI
~BAC
HR
AmpR
HR
3
3
+
FLP plasmid
FLP mediated excision of the 3’ targeting construct
CAG
PGK
NeoR
GOI
~70kb BAC
3
CHO wt
plasmid
C
B
42.7% GFP positive
0.0% GFP positive
### Chart
| Category | |
|---|---|
| CHO WT | 0.0 |
| plasmid | 17.08 |
| 210 kb BAC | 4.13 |
| 70 kb BAC | 11.99 |70kb BAC
210kb BAC
88.3% GFP positive
99.3% GFP positive
Supplementary Figure 4. Generation of the truncated 70kb BACRosa26 and comparison of the transfection efficiency and expression level of the two BACRosa26
(A) Experimental outline of the truncated 70kb BACRosa26 generation. The original BACRosa26 was shortened by homologous recombination and FLP mediated excision of the targeting constructs. (B) Nucleoporation efficiency of different CAG promoter-GFP constructs. Ratio of GFP positive cells was measured by FACS in transfected CHO cells 24 hours after nucleoporation. (C) GFP fluorescence intensity of stable CHO cell pools was measured 2 months after nucleoporation with constitutive G418 selection.
