# Supplementary material for: High-throughput ligand profile characterization in novel cell lines expressing seven heterologous insect olfactory receptors for the detection of volatile plant biomarkers
Source: Sci Rep. 2023 Dec 8;13:21757. doi: 10.1038/s41598-023-47455-4 (PMC10709440; doi:10.1038/s41598-023-47455-4)
Supplement: Supplementary file 2 — Supplementary Table 1. [file 41598_2023_47455_MOESM2_ESM.pdf]

Supplementary Table 1.

Primer list for cDNA cloning of olfactory receptor (Or) genes

Name	Sequence (5’-3’)
Or10a for	TTTTTGGCGCGCC**GCCACCATG**TCCGAGTGGTTACGC
Or10a rev	AAAAAGGCGCGCCTTACTGAAAGGACTTAACCAGC
Or13a for	TTTTTGGCGCGCC**GCCACCATG**TTCTATTCGTATCCCTAC
Or13a rev	AAAAAGGCGCGCCTTAATCTAGTTTCTTTTCGTCG
Or19a for	TTTTTATTTAAAT**GCCACCATG**GACATATCGAAGGTGG
Or19a rev	AAAAAATTTAAATCTATTCAAGGGACGTTTTACG
Or47b for	TTTTTGGCGCGCC**GCCACCATG**AACGACTCGGGTTATC
Or47b for	AAAAAGGCGCGCCCTACATCGATTCTTGCATCAG
Or49b for	TTTTTGGCGCGCC**GCCACCATG**TTTGAAGACATTCAGC
Or49b rev	AAAAAGGCGCGCCTCATCCGTAGACTCGCTT
Or67b for	TTTTTGGCGCGCC**GCCACCATG**CAGGACCAACTGGATC
Or67b rev	AAAAAGGCGCGCCCTATTTGTTGTTCATGTTGCG
Or69a for	TTTTTGGCGCGCC**GCCACCATG**CAGTTGCACGACCATATG
Or69a rev	AAAAAGGCGCGCCTTATTTAAGGGACCGCACAC
Or71a for	TTTTTGGCGCGCC**GCCACCATG**GACTACGATCGAATTC
Or71a rev	AAAAAGGCGCGCCCTATTGGTTCATGTTGAGC
Or85b for	TTTTTGGCGCGCC**GCCACCATG**GAGAAGCTAATGAAGTAC
Or85b rev	AAAAAGGCGCGCCCTATTGGGTATACATTGTGC
Or98a for	TTTTTGGCGCGCC**GCCACCATG**TTGTTCAACTATCTGCG
Or98a rev	AAAAAGGCGCGCCTCAGTTCTTTGTCAATCTGTC

Restriction enzyme recognition sites (*Swa*I for OR19a, *Asc*I for all other receptors) underlined, Kozak consensus sequences in **bold**.

Primer list for the generation of the 70 kb Rosa26 BAC targeting constructs

Name	Sequence (5’-3’)
BAC $^{Rosa26\ 70kb}$ 5’ for	GAATTTTCTATATTATGAATGTCTCTGTAATAAATAAATCAATTCTTCAAGCGAAG<br>TTCCTATTCTCTAGAAAG
BAC $^{Rosa26\ 70kb}$ 5‘ rev	GATAAATACGTACTTCAAGTTTAAGAGTGAGAGAAACTTCAAGGCAGTTCAGAA<br>GTTCCTATACTTTCTAGAG
BAC $^{Rosa26\ 70kb}$ 3‘ for	TGAACCATCTTGCTAACTCACAGTGGTATTTTCTCAGGATACTTCTGTTTGAAGTT<br>CCTATTCTTCAAATAG
BAC $^{Rosa26\ 70kb}$ 3‘ rev	TGCCTCAGGATGTCTTGATTTTGGTTATCTGGGATTAAATCTACTAAGTATGGAA<br>GTTCCTATACTATTTGAAG
